# The “Alluvial Mesovoid Shallow Substratum”, a New Subterranean Habitat

**DOI:** 10.1371/journal.pone.0076311

**Published:** 2013-10-04

**Authors:** Vicente M. Ortuño, José D. Gilgado, Alberto Jiménez-Valverde, Alberto Sendra, Gonzalo Pérez-Suárez, Juan J. Herrero-Borgoñón

**Affiliations:** 1 Departamento de Ciencias de la Vida, Facultad de Biología Ciencias Ambientales y Química, Universidad de Alcalá, Alcalá de Henares, Madrid, Spain; 2 Departamento de Biología Animal, Facultad de Ciencias, Universidad de Málaga, Málaga, Spain; 3 Departamento de Botánica, Facultad de Ciencias Biológicas, Universidad de Valencia, Burjassot, Valencia, Spain; 4 Departamento de Biogeografía y Cambio Global, Museo Nacional de Ciencias Naturales, Madrid, Spain; Roehampton University, United Kingdom

## Abstract

In this paper we describe a new type of subterranean habitat associated with dry watercourses in the Eastern Iberian Peninsula, the “Alluvial Mesovoid Shallow Substratum” (alluvial MSS). Historical observations and data from field sampling specially designed to study MSS fauna in the streambeds of temporary watercourses support the description of this new habitat. To conduct the sampling, 16 subterranean sampling devices were placed in a region of Eastern Spain. The traps were operated for 12 months and temperature and relative humidity data were recorded to characterise the habitat. A large number of species was captured, many of which belonged to the arthropod group, with marked hygrophilous, geophilic, lucifugous and mesothermal habits. In addition, there was also a substantial number of species showing markedly ripicolous traits. The results confirm that the network of spaces which forms in alluvial deposits of temporary watercourses merits the category of habitat, and here we propose the name of “alluvial MSS”. The “alluvial MSS” may be covered or not by a layer of soil, is extremely damp, provides a buffer against above ground temperatures and is aphotic. In addition, compared to other types of MSS, it is a very unstable habitat. It is possible that the “alluvial MSS” may be found in other areas of the world with strongly seasonal climatic regimes, and could play an important role as a biogeographic corridor and as a refuge from climatic changes.

## Introduction

For nearly eight decades since the appearance of biospeleology as a scientific discipline following the publication of the study by Racovitza [1], classic authors such as Jeannel [2,3], Vandel [4] or Ginet & Decou [5] have been drawing attention to the extent of the subterranean domain in cracks and fissures beneath the soil surface (microcaverns and mesocaverns sensu Howarth [6]), besides the caves accessible to humans. However, it was not until the 1980s that the studies led by Christian Juberthie [7,8] and the observations carried out by Shun-Ichi Uéno [9,10] revealed the extent of the subterranean domain beneath the karst surface shallow, designated Milieu Souterrain Superficiel in French, and later translated to English as Mesovoid Shallow Substratum, abbreviated to MSS. It forms a unique habitat with its own fauna, but can also play the role of an ecotone, in which both epigean and truly subterranean, troglobiont life forms thrive. A continuum of life is established from the surface soil, through the different edaphic horizons and the MSS, and finally, in the deep subterranean environment, as reported in a study by Gers [11].

A basic condition for the existence of the MSS is the presence of rocky deposits that have generated - and preserved - subterranean spaces which life forms can inhabit. There are many diverse forms of the MSS habitat, which arise as the result of several abiotic and biotic factors [12]. To date, three basic types of MSS have been distinguished:

### 1): Colluvial or slope MSS

This is formed on sloping ground [7], and is the result of the erosion and deposition of rock fragments produced by the mechanical fragmentation of rock walls or the erosive action of continental glaciers. The nature of the rock can be diverse, including calcareous, siliceous and even volcanic rocks. These rocky deposits can be covered by evolving soil (with edaphic horizons) of different thicknesses which often supports dense plant cover. In other cases, the upper level of the MSS is bare, appearing as mountain scree.

### 2): Bedrock MSS

This is formed by the weathering of much of the bedrock [13], a gradual process that occurs almost simultaneously with the formation of edaphic horizons which seal it off from the external environment. This type of MSS is found in valley bottoms or areas with little or no slope. The nature of the rock may be very diverse, although it is more likely to occur in rocks that are easily altered.

### 3): Volcanic MSS

This is the result of the accumulation of volcanic material [14]. From formation onwards, it contains many interconnected fissures forming a tangled web of micro-spaces, which over time become isolated from the outside due to the development of an edaphic environment [15]. This type of MSS is more ephemeral than others, since deposits of volcanic material are more susceptible to weathering than those composed of more compact rocks such as sedimentary, plutonic or metamorphic rocks.

In this paper, we present a proposal supported by experimental and theoretical arguments for a new type of MSS which we have named “alluvial MSS”, and which corresponds to the crevices (unfilled spaces) which lie beneath the streambeds of temporary watercourses.

## Antecedents

The existence of hypogean species in watercourses was already known from occasional citations [16,17], but it was the search for *Thalassophilus breuili* Jeannel, 1926, endemic to Alicante, that triggered the study which led us to propose a new type of MSS habitat, the “alluvial MSS”. Taking into account that this species was so rare in caves (only three specimens from two caves are known [18,19]) and that the third time it was cited, it was on the banks of a temporary watercourse (the Guadalest River) in Altea (Alicante, Eastern Spain) [17], this suggested the possibility that this species might have been swept away by water from a nearby MSS, and that it might be found in this latter habitat [20]. Given the extraordinary irregularity of the hydrological regime of watercourses in the province of Alicante, it was thought that perhaps *T. breuili* might live in the substratum of these streambeds. Pursuing this lead, one of the authors (V.M.O.) installed 4 pitfall traps, buried 50-70 cm deep, in the streambed of the watercourse known as the “*Barranco de la Cueva de los Corrales*” (Xixona, Alicante, Spain). These traps were operated for 4 months (June-September, 2009), collecting a considerable number of arthropods, which abounded in the interstices of the substratum of the watercourse. Although *T. breuili* was not found, it is worth noting the capture of more than a hundred *Speonemadus* sp., Coleoptera Leiodidae typical of large subterranean cavities and MSS [21,22]. All these data prompted the launch of a research project aimed, among other objectives, at prospecting and studying the subterranean spaces located among the alluvial deposits of dry watercourses in the province of Alicante. These watercourses are dry for most of the year, carrying water only during very sporadic periods of heavy rainfall. However, this subterranean environment remained damp throughout the year and a priori, its structure, comprising stony debris with an extensive network of crevices, could provide a refuge for terrestrial stenohygrobic fauna.

## Experimental approach

### Study area

The geographic area selected for our study encompassed the eastern end of the karst reliefs of the Prebetic range, from the centre and north of the province of Alicante to the south of the province of Valencia (Eastern Spain), and was chosen because there are numerous temporary watercourses in the area, thanks to a series of geological, geographical and climatic factors. In the local toponymy, these types of watercourse are termed “*rambla*” (low, wide channel) and “*barranco*” or “*barranc*” (tall, narrow channel), words which reflect cultural and linguistic factors [23,24] rather than a true hydrographic characterisation. Regardless, these dry watercourses are usually dry for most of the year, only carrying water for an average of ten days a year [24,25].

**Figure 1:**
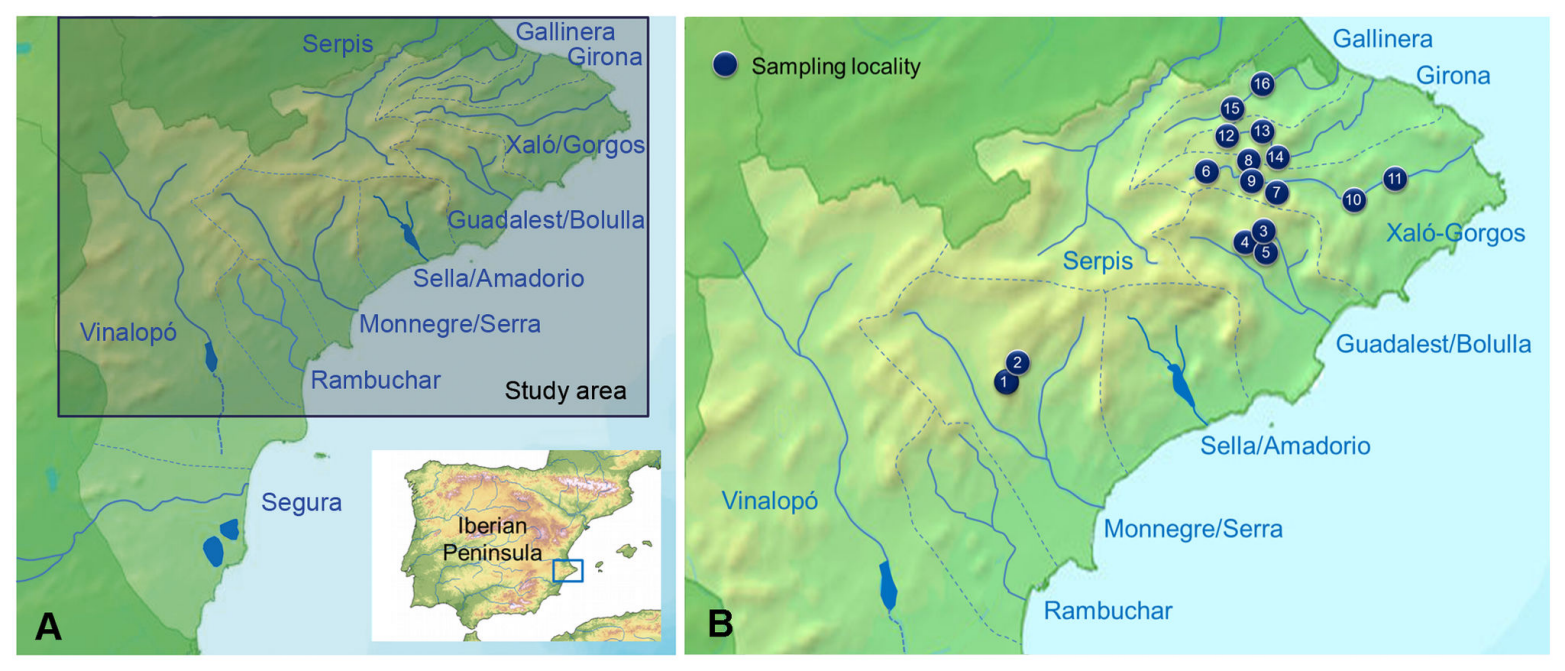
Study area. (A) Alicante watersheds. (B) Sampling points.

The study area was divided into first-order river basins (Figure 1), following the criterion of IDEJúcar [26], and subsequently, basins in the northern half, the wettest, most mountainous area, were selected. A preliminary selection of promising sites for sampling was made using Sigpac [27] and Google, Earth [28]. Of the 42 sites selected a priori, 16 were considered eligible (Figure 1; Table 1) because the watercourses they contained were dry for most of the year and had thick alluvial deposits with numerous subterranean spaces. The other sites were rejected because they did not present either of the two features mentioned. Many of them overlay inappropriate lithologies, as was the case with the marly series of the Tap [29] which constitute physical barriers to the movement of subterranean fauna [30].

**Table 1:** Selection of locations for the study of “alluvial MSS” in the province of Alicante.

WATERSHED	LOCALITY	ALTITUDE m.s.n.m.	UTM COORDINATES DATUM WGS 84 (30 S)	SSD DEPTH (cm)
Monnegre-Serra	1 Barranco de la Cueva de los Corrales	752	714792 4271389	50
	2 Barranc dels Ports	707	715155 4272106	100
Guadalest-Bolulla	3 Barranc del Xarquet	537	749378 4288403	50
	4 Barranc de Sacanyar	704	747654 4287890	75
	5 Río Bolulla	310	750557 4286654	50
Xaló-Gorgos	6 Barranc de Famorca	613	740688 4290552	100
	7 Barranc de Almadich	500	750272 4291758	75
	8 Barranc de Malafí	478	744002 4294237	50
	9 Río Castells	449	744750 4292415	100
	10 Barranc de Masserof	230	759899 4291000	50
	11 Río Xaló	103	243542 4294802	75
Girona	12 Barranc d’Alcalà	560	741444 4298843	75
	13 Barranc de Turrubanes	394	745211 4299809	75
	14 Barranc de Cocons	407	746090 4298420	65
Gallinera	15 Barranc de la Vall de Gallinera	216	744050 4302706	75
	16 Barranc de la Vall de Gallinera	160	744991 4303533	50

The permissions for the sampling and capture of invertebrate fauna in the study area were given by the Conselleria d’Infraestructures, Territori i Medi Ambient of the Generalitat Valenciana. No private lands or protected areas were sampled, and neither endangered nor protected species were involved in this study. Specimens collected are deposited in the zoological collection of the University of Alcalá.

### Construction and installation of subterranean devices

**Figure 2:**
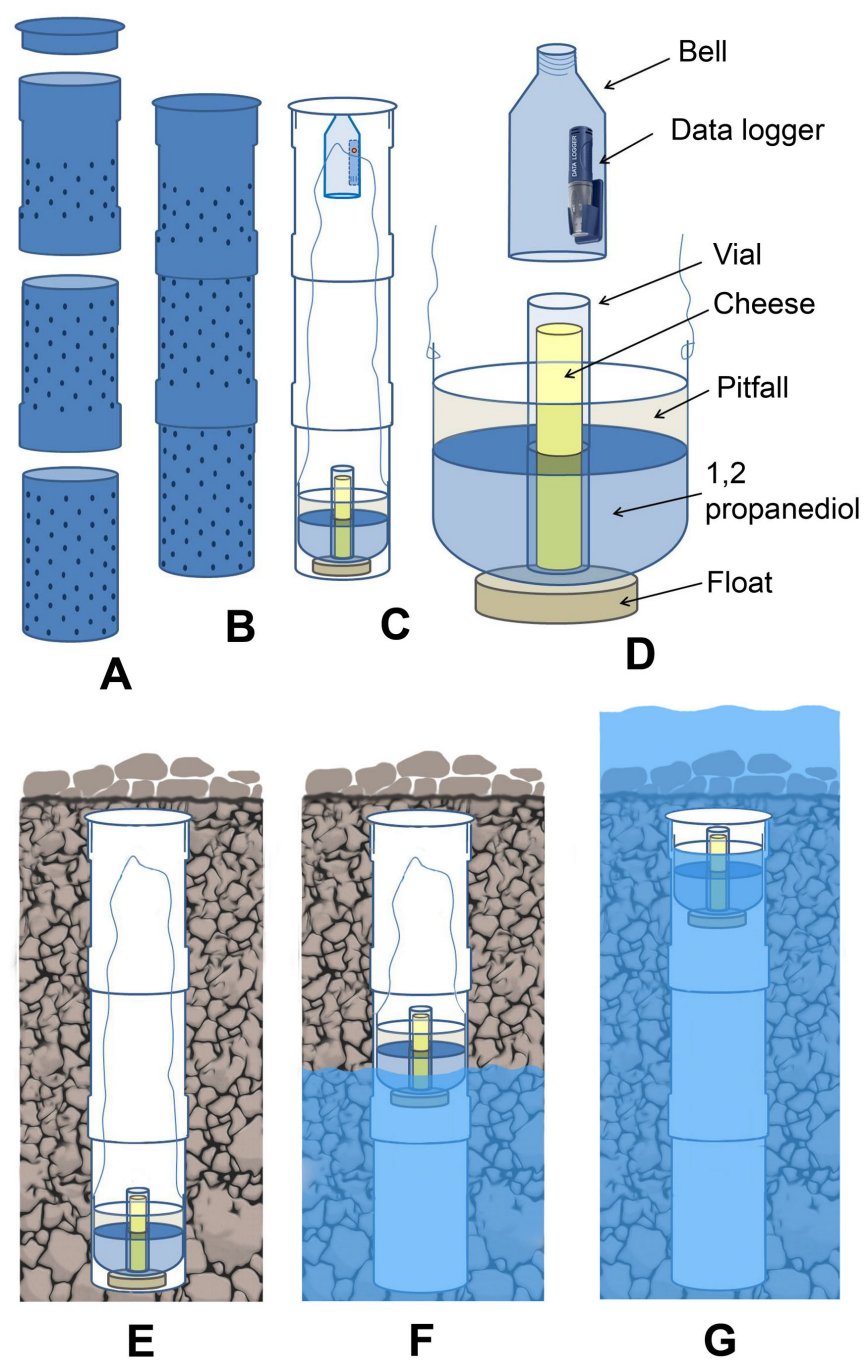
Subterranean Sampling Device design. (A) Cover and interlocking tube sections to accommodate different substrate thicknesses. (B) Assembled tube sections. (C) Longitudinal tube section showing internal devices (data collection and data loggers). (D) Detail of the capture devices (pitfall trap with float to avoid submersion in the case of a temporary rise in the phreatic level) and the digital temperature and humidity sensor (hourly data recording) protected inside a plastic hood. (E-F) Operation of the auto-save device should the watercourse become active again due to a gradual increase in the phreatic level.

To avoid mechanical disturbance of the substratum each time the pitfall traps were removed, a multi-perforated cylinder was previously buried underground; the perforations gave fauna access to the inside whilst the cylinder enabled their subsequent collection since the pitfall trap could be removed via the vertical tube. This technique was first invented by Gers [11,13] and later developed by Owen [31]. Several years later, López & Oromí [32] proposed a somewhat more sophisticated trap model. Henceforth, we will refer to these devices as “subterranean sampling devices” (SSD). The design of the SSD used in this study was based on the model described by López & Oromí [32] to which some innovations were added (Figure 2). Cylinder length varied between 50 and 100 cm, and the cylinders were perforated below the upper 20 cm. The pitfall trap was lowered into and retrieved from the bottom of the tube using a nylon cord. The fauna collection chamber contained 1,2-propanediol, which does not evaporate and enables preservation of DNA. It also had a small central compartment which housed a phial, in the form of a renewable cartridge, containing solid bait (very strong-smelling cheese) to attract arthropods [4]. A single SSD was installed in each chosen location. The traps inside each SSD were operated for 12 months and were renewed every three months, from October 2011 to October 2012. Temperature readings were taken both inside the cylinders (at a depth of 20-30 cm) and above ground (at a height of between 1 and 2 m), recording data every hour (in most cases) or every ½ hour. Mean (T_mean_), minimum (T_min_) and maximum (T_max_) temperatures were calculated for the entire cycle, as was the mean of the minimum (T_Mmin_) and maximum (T_Mmax_) daily (24 hours) temperature and the mean of the daily temperature range (T_Dmean_). We applied a filter (moving average) to the time series using a window size of 12 (in most cases) or 24 hours, in order to better visualise the temperature pattern over time and determine the day/night cycle; minimum (T_Fmin_) and maximum (T_Fmax_) temperatures were calculated after applying the filter. We also measured relative humidity inside the cylinders and calculated the mean (RH_mean_), minimum (RH_min_) and maximum (RH_max_) for the entire cycle.

## Results

### Relative humidity and temperature

**Figure 3:**
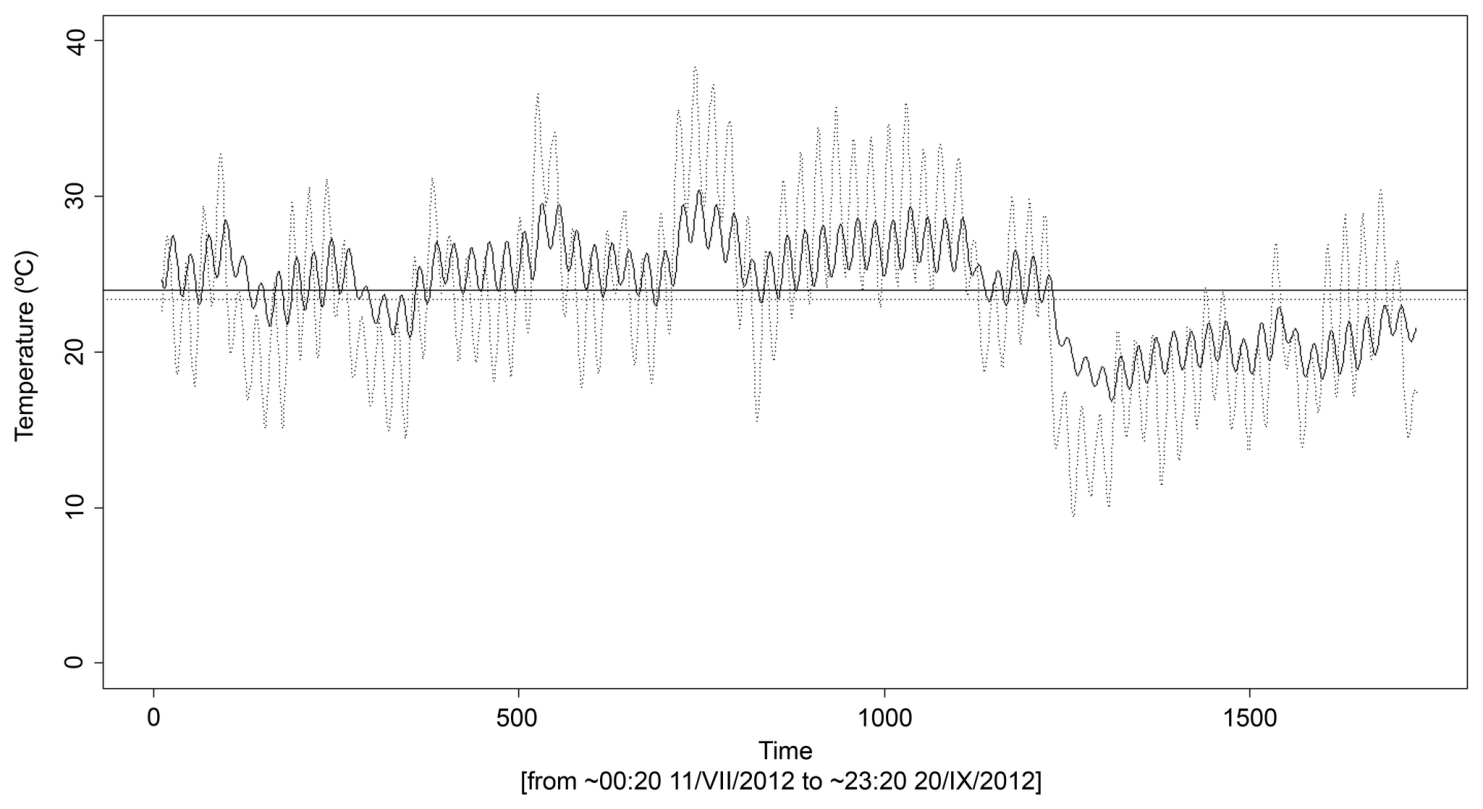
Temperature series in Barranco de la Cueva de los Corrales (Subterranean Sampling Device 1). Data for the interior of the Subterranean Sampling Device (solid line) and above ground (dotted line) after applying linear filtering (moving average) with a 12-hour window size. Horizontal lines represent mean values (solid line, Subterraenan Sampling Device; dotted line, above ground). The graph shows how the temperature inside the SSD was not as extreme as above ground and how the daily cycles were slightly out of phase.

Due to unpredictable floods, most of the data loggers were lost or spoilt and no data was obtained for a complete annual cycle; hence, the results given here are partial and they are thus impossible to generalise. Partial data for both the SSDs and the external environment were recovered for three locations, whilst for a fourth location, only SSD data were recovered. RH values inside the SSDs almost always showed complete saturation, with RH_mean_ values which were always above 80% and presented little variation (Table 2). With regard to temperature, T_mean_ values inside the SSDs and above ground were similar (Table 2), and SSD temperatures reflected both the daily and the seasonal cycle of the external environment, although the temperature cycle was somewhat out of phase since maximum and minimum temperatures were reached a few hours later in the substratum (Figure 3). The range of temperature variation, both for the entire cycle ([T_max_-T_min_] and [T_Fmax_-T_Fmin_]) and the daily cycle (T_Dmean_), was much lower in the SSD than above ground (Figure 3 and Table 2). As regards minimum temperature values, although T_min_ values in the MSS occasionally fell slightly below 0°C in February, T_Mmin_ values rarely fell below 8°C (Table 2). T_Mmin_ values in the MSS were always above the values recorded above ground and showed much less variation. In contrast, T_Mmax_ values in the MSS were always below those above ground, and also showed less variation. The maximum temperature in the SSDs did not exceed 31.8°C even though temperatures above ground reached as high as 44.3°C, whilst the maximum T_Mmax_ value recorded for the MSS was 26.55°C.

**Table 2:** Temperature and relative humidity data for the interior of the SSDs and above ground.

Locality		Dates	Time step	RH_mean_ (CV)	RH_min_/RH_max_	T_mean_ (CV)	T_Dmean_	T_min_/T_max_ (T_Fmin_/T_Fmax_)	T_Mmin_/T_Mmax_ (CV)
Barranco de la Cueva de los Corrales	Surface	11/VII/2012- 20/IX/2012	1 hour	−	−	23.36 (29.33)	14.35	7.4/44.3 (9.37/38.3)	16.81/31.16 (25.94/18.02)
	SSD			80.53 (14.36)	46.5/100	23.98 (13.57)	4.96	16/31.8 (16.84/30.4)	21.58/26.55 (11.79/11.73)
	Surface	10/X/2011- 09/XI/2011		77.35 (24.35)	−	14.02 (32.28)	10.79	7/33 (7.41/24.97)	9.86/20.65 (20.49/28.11)
	SSD			93.57 (6.77)	65.5/100	13.39 (17.22)	2.33	6.3/18.8 (8.08/17.92)	12.25/14.58 (16.90/16.53)
Barranc de Cocons	Surface	11/X/2011- 25/XI/2011		81.84 (20.82)	−	14.03 (34.84)	11.75	4.7/35.7 (5.84/25.93)	9.54/21.29 (23.35/28.78)
	SSD			97.57 (3.97)	70.4/100	14.99 (12.27)	1.12	11.9/18.9 (12.27/18.7)	14.35/15.46 (12.18/12.60)
Barranc de la Vall de Gallinera	Surface	10/X/2011- 07/I/2012		77.18 (24.65)	−	13.28 (45.19)	14.78	0/39.1 (1.42/27.27)	8.08/22.85 (42.71/26.81)
	SSD			99.49 (1.95)	83.1/100	14.07 (22.04)	3.21	5.2/21.2 (6.01/20.47)	12.68/15.89 (24.98/14.89)
Barranc de Almadich	SSD	28/X/2011- 07/V/2012	½ hour	99.43 (1.91)	86.5/100	9.03 (40.15)	1.77	−0.3/17 (−0.20/16.74)	8.28/10.05 (45.54/33.42)

Mean temperature (T_mean_); minimum temperature (T_min_); maximum temperature (T_max_); mean of the minimum (T_Mmin_) and maximum (T_Mmax_) daily (24 hours) temperature; mean of the daily temperature range (T_Dmean_). Minimum (T_Fmin_) and maximum (T_Fmax_) temperature after applying a linear filter (moving average) to the time series with a window size of 12 (in the cases of 1 hour time steps) or 24 (in the cases of ½ hour time steps); mean relative humidity (RH_mean_); minimum relative humidity (RH_min_); maximum relative humidity (RH_max_).

### Fauna results

The initial taxonomic studies of the samples highlighted the ecological importance of the dry watercourse substratum for terrestrial fauna (Table 3, Table S1). The specimens collected were unevenly distributed among different phyla, both quantitatively and qualitatively. In cases where the SSDs had been affected by a rise in the phreatic level, or the watercourse had recovered its ephemeral hydrological activity, specimens of aquatic or semi-aquatic life were collected, belonging to the phyla Rotifera, Plathelminthes (class Turbellaria), Mollusca (class Gastropoda), Tardigrada, Arthropoda (classes Maxillopoda, Ostracoda, Malacostraca and Insecta) and Chordata (class Amphibia). Despite obtaining data for aquatic fauna, the majority of the fauna collected was terrestrial since these watercourses are dry for most of the year. These terrestrial specimens belonged to the phyla Mollusca (Class Gastropoda), Annelida (class *Oligochaeta*), Nematoda and Arthropoda, with a predominance of the latter group. Among the terrestrial Arthropoda in the substratum of these watercourses, the four current subphyla were well represented. Of note among the Chelicerata, and more specifically the Arachnida, was the presence of the Acari, especially of groups such as the Oribatida and the Gamasida, most of which can be considered edaphic species [33] which invade subterranean spaces. The Opiliones contributed several species, among which can be highlighted *Trogulus lusitanicus* Giltai, 1931 and *Dicranolasma soerensenii* Thorell, 1876. The greater abundance of the latter species in the substratum of the watercourses may be due to its troglophile lifestyle [34], as it has often been observed in caves where it appears to complete its life cycle [35] and has also been found in the colluvial MSS in the study area (unpublished data). Other Arachnida that appeared regularly in alluvial substratum samples were the Pseudoscorpiones, a group which typically includes numerous species specialising in the subterranean environment of the eastern reliefs of the Prebetic System [36,37]. Especially conspicuous among the Araneae were the Linyphiidae family, and in particular, the genus *Lepthyphantes* Menge, 1866 (*sensu lato*), a group that numbers many species closely associated with the subterranean environment [38,39]. The Linyphiidae *Lessertia dentichelis* (Simon, 1884) was also well represented, a species with a high preference for damp areas and a habitual inhabitant of both natural and artificial cavities in the Mediterranean region [40]. The species *Pardosa* cf. *tatarica* (Thorell, 1875) (Lycosidae) is closely associated with sandy places and streams edges [41]. Although *Pardosa* C. L. Koch, 1847 is a clearly epigean genus [41,42], the sampling results suggest that an appreciable population of P. cf. *tatarica* inhabits the deeper levels of the streambed, but are rarer on the surface since the watercourses remain completely dry for most of the year (unpublished data). Something similar has been observed with spiders of the genus *Dysdera* Latreille, 1804 (Dysderidae). Above ground, whether dwelling under rocks or in plant detritus [43], these spiders are extremely rare in this Mediterranean area. However, they are much more abundant in the substratum, having been observed not only in alluvial substrata but also occasionally in caves in the Eastern Prebetic range [44,45] and in many colluvial MSS in the study area (unpublished data). This tendency towards a certain level of hypogean activity coincides with the abundance of terrestrial Isopoda (suborder Oniscidea) in this type of habitat. The Myriapoda also had a strong presence in the substratum of the dry watercourses, with Diplopoda from the Polydesmida and, to a lesser extent, the Glomerida and Callipodida orders being particularly numerous. This behaviour may be due to the lucifugous and hygrophilous nature of these arthropods [46] which, similar to the Crustacea Isopoda, find the conditions of epigean environments more hostile. The Chilopoda appeared frequently in the alluvial substratum, among which we found specimens of the genus *Lithobius* Leach, 1814, which is also common in other subterranean spaces, with a well-documented presence in caves in the Iberian Peninsula [39]. Other Chilopoda captured in the alluvial substratum belonged to the genera *Cryptops* Leach, 1814, *Scolopendra* Linnaeus, 1758, and two very characteristic species of Western Mediterranean areas, *Theatops erythrocephala* (C.L. Koch, 1847) and *Scutigera coleoptrata* (Linnaeus, 1758). The Hexapoda presented a wide diversity of species in the alluvial substratum samples, and we observed specimens of the following orders: Collembola, Archaeognatha, Zygentoma, Diplura, Dyctioptera, Dermaptera, Orthoptera, Embioptera, Psocoptera, Hemiptera, Coleoptera, Diptera, Lepidoptera and Hymenoptera. Of these, two orders will be discussed in detail here, for which some interesting data were obtained. The Orthoptera contributed species from the genus *Petaloptila* Pantel, 1890 to the ensemble of fauna inhabiting the substratum of these watercourses. These Gryllidae exhibit troglophilic habits [39], which would explain their presence in these subterranean environments of fluvial origin as well as in other subterranean spaces in the region, such as caves [47]. The Coleoptera present a wide diversity of species and, as in other subterranean environments, the Carabidae are one of the most representative families of hypogean fauna in the Iberian Peninsula [48]. Of particular note was the presence of specimens of *Trechus* Clairville, 1806 belonging to the lineage *T. martinezi* (sensu Ortuño & Arillo, 2005), previously only described in a few cave environments [49] and on isolated occasions in the bedrock MSS of the Bernia mountain range in the province of Alicante [50]. We also found *Porotachys bisulcatus* (Nicolaï, 1822), a stenohygrobic and lucifugous species that in much of the Iberian Peninsula behaves as a true troglophile [51], and *Ocys harpaloides* (Audinet-Serville 1821), which is eurytopic and mesothermal, living in a variety of damp, dimly lit or completely dark habitats and capable of developing troglophilic habits [52]. In addition, we regularly collected numerous specimens of a Carabidae species of the genus *Bembidion* Latreille, 1802 which, in the dry watercourses in this geographic area, has not been observed either as a riparian sub-lapidicole or moving over the surface of the streambed. It is also worth noting the presence of *Platyderus* Stephens, 1827 in the alluvial substratum, a genus containing geophilic and lucifugous species with sub-lapidicole habits which also exhibit a clear tendency to occupy subterranean spaces [53]. The Leiodidae is another family that is well established in hypogean environments [22,54], and the genus *Speonemadus* Jeannel, 1922 was particularly numerous in the samples collected. In general, the species of this genus are known to inhabit subterranean environments, especially caves and the MSS [21]. Also striking was the recurrent presence of Lampyridae larvae (genera *Nyctophila* Olivier, 1884 and *Lamprohiza* Motschulsky, 1853) which, given their helicophagy [55] must have been on the tracks of the Mollusca Gastropoda that inhabit the damp interstices of the substratum. Although Lampyridae larvae have been observed in entrance zone of caves [30], their presence in the MSS has not been described for any Lampyridae species in the Iberian Peninsula [56,57].

**Table 3:** Number of specimens of some representative taxa of the “alluvial MSS”.

Taxa	Number of individuals in each locality (1-16) of “alluvial MSS”															
	1	2	3	4	5	6	7	8	9	10	11	12	13	14	15	16
Opiliones																
*Dicranolasma soerensenii* Thorell, 1876	−	−	−	10	1	−	3	25	−	31	−	−	−	−	−	−
*Trogulus lusitanicus* Giltai, 1931	−	−	−	2	−	−	1	12	−	1	−	−	1	3	−	−
Araneae																
*Lepthyphantes* (*sensu lato*) spp.	4	8	−	2	7	2	11	1	−	4	−	−	2	−	−	2
*Lessertia dentichelis* (Simon, 1884)	−	−	−	−	6	−	−	−	1	−	1	12	−	−	−	−
*Pardosa* cf. *tatarica* (Thorell, 1875)	−	−	7	−	−	−	2	−	3	−	2	−	−	−	−	−
*Dysdera* sp.	1	−	−	−	−	−	−	−	1	−	−	1	−	−	1	1
Lithobiomorpha																
*Lithobius lusitanicus* Verhoeff, 1925	−	−	−	−	−	−	−	−	1	−	2	−	−	−	−	1
*Lithobius castaneus* Newport, 1844	1	2	2	−	2	−	−	11	−	1	−	−	−	1	−	1
*Lithobius* sp.	−	−	−	−	−	1	−	−	−	−	−	−	1	−	−	−
Scolopendromorpha																
*Cryptops hispanus* Brolemann, 1920	−	−	−	−	2	−	−	−	−	−	−	−	−	−	−	−
*Scolopendra cingulata* Latreille, 1789	−	−	−	−	−	−	−	1	1	−	1	−	−	−	−	−
*Scolopendra oraniensis* H. Lucas, 1846	1	−	−	−	−	−	−	−	−	−	−	−	−	−	−	−
*Theatops erythrocephala* (C.L. Koch, 1847)	−	−	−	−	−	1	9	13	−	−	−	−	−	−	−	1
Scutigeromorpha																
*Scutigera coleoptrata* (Linnaeus, 1758)	−	−	1	−	−	4	−	3	2	1	1	−	4	−	−	3
Orthoptera																
*Petaloptila* (*Petaloptila*) *aliena* (Brunner-Wattenwyl, 1882)	−	23	−	5	2	−	2	−	19	−	3	2	4	1	−	−
*Petaloptila* (*Zapetaloptila*)*bolivari* (Cazurro, 1888)	−	−	−	2	2	−	2	−	10	−	2	−	−	−	−	−
Coleoptera																
*Trechus* n. sp. (“*T. martinezi* lineage”)	−	−	−	1	21	−	−	3	−	−	−	−	1	−	−	−
*Porotachys bisulcatus* (Nicolaï, 1822)	−	−	−	2	1	−	−	−	−	−	−	−	1	9	−	−
*Ocys harpaloides* (Audinet-Serville, 1821)	−	−	−	−	−	−	−	−	1	−	1	−	8	−		−
*Bembidion* (*Ocyturanes*) *martachemai* (Toribio, 2002)	16	16	11	7	27	4	83	1	9	20	9	29	23	11	−	−
*Platyderus* sp.	7	−	−	−	1	−	2	−	−	1	−	−	−	−	−	−
*Speonemadus escalerai* (Uhagon, 1898)	158	387	−	−	−	−	−	−	−	−	−	−	−	−	−	−
*Nyctophila reichii* (Jacquelin du Val, 1859)	−	3	−	−	−	−	−	−	3	6	4	−	1	−	−	−
*Lamprohiza* sp.	−	1	−	1	1	1	1	1	−	2	−	−	−	−	−	−

Representative taxa of the “alluvial MSS” (see text for details). The specimens were collected over a year of sampling in each of the SSDs installed in the province of Alicante. The number assigned to each location corresponds to that given in Table 1.

### Description of a New Subterranean Habitat: The “alluvial MSS”

For the most part, the fauna communities found in the alluvial substratum consisted of species belonging to four groups with well-defined ecophysiological characteristics, being geophilic, hygrophilous, mesothermal and/or lucifugous species. These species exhibit a geophilic nature, inhabiting environments closely associated with soil (edaphic horizons or different epiedaphic habitats and biotopes). They can be either totally geophilic, i.e. their entire life cycle occurs in these habitats/biotopes, or partially geophilic, in which only the larval stages inhabit this type of environment, as in the case of Lampyridae species of the genera *Nyctophila* and *Lamprohiza*, both frequently observed in the alluvial substratum. The second ecophysiological characteristic is the strongly hygrophilous nature of the species which were well represented in the alluvial substratum. In areas such as that studied, with a Mediterranean climate, these highly hygrophilous species are extremely rare or absent in edaphic/epiedaphic environments. The third characteristic is the mesothermal nature of many of these species, a circumstance that forces them to seek refuge in favourable environments which mitigate the sudden daily and/or seasonal changes in temperature. The last ecophysiological characteristic was the lucifugous nature which, however, only a portion of the ensemble of alluvial substratum fauna exhibited. This characteristic may be expressed to a lesser or greater degree, and thus some species may show a preference for shaded environments (sciophily) whilst others thrive in aphotic environments (a diversity of subterranean environments).

Different species presented different combinations of these characteristics. There were strongly hygrophilous, lucifugous and mesothermal species, all found in caves and other subterranean spaces, such as species belonging to the lineage *Trechus martinezi*, *Lepthyphantes* spp. and certain Isopoda Oniscidea, among others, which are considered troglobiont forms. Then there were strongly hygrophilous, mesothermal and sciophilic species (not exclusive to aphotic environments), such as *Dysdera* spp., *Lithobius* spp., *Porotachys bisulcatus*, *Ocys harpaloides*, *Platyderus* spp., *Speonemadus* spp. and some Oniscidea Isopoda, among others, which constitute an ensemble of species which are typically troglophilic. There were also moderately hygrophilous, mesothermal and sciophilic species which may sometimes behave like true troglophiles, such as *Dicranolasma soerensenii* and *Petaloptila* spp., and lastly, there were species which were markedly hygrophilous, may or may not be mesothermal and were not lucifugous, as is the case of fauna exhibiting ripicolous behaviour such as *Pardosa* spp. and some of the Carabidae Bembidiinae species, among others.

Thus, as happens in caves, the alluvial substratum constitutes a habitat occupied by fauna which is exclusively hypogean (troglobionts), frequently hypogean (troglophiles), and where numerous edaphic species occur (Acari and Collembola, among others) to a different degree, in addition to ripicolous and trogloxene fauna. The latter group consisted of species with a small number of individuals belonging to different orders (Diptera and Hymenoptera, among others), for which living conditions in the substratum are not optimal. This group of specimens reached the substratum by accident, either actively, as part of their daily scavenging activities in the edaphic/epiedaphic environment, or passively, usually as a result of hydrochory and also zoochory. Despite being considered species which are not adapted to this environment, they make a vital contribution to the flow of energy in these subterranean habitats [11,58]. Therefore, there is fauna with different ecological roles: some of them shared with caves and other MSSs (troglobionts and trogloxenes), which characterizes this habitat as a true MSS; some others are typical of soil (edaphobionts), which are also frequent in other MSS [59]; epigean fauna (trogloxenes), due to its proximity to the surface and the conditions of temperature and humidity; and finally ripicolous fauna, whose presence would be particularly characteristic of this habitat.

**Figure 4:**
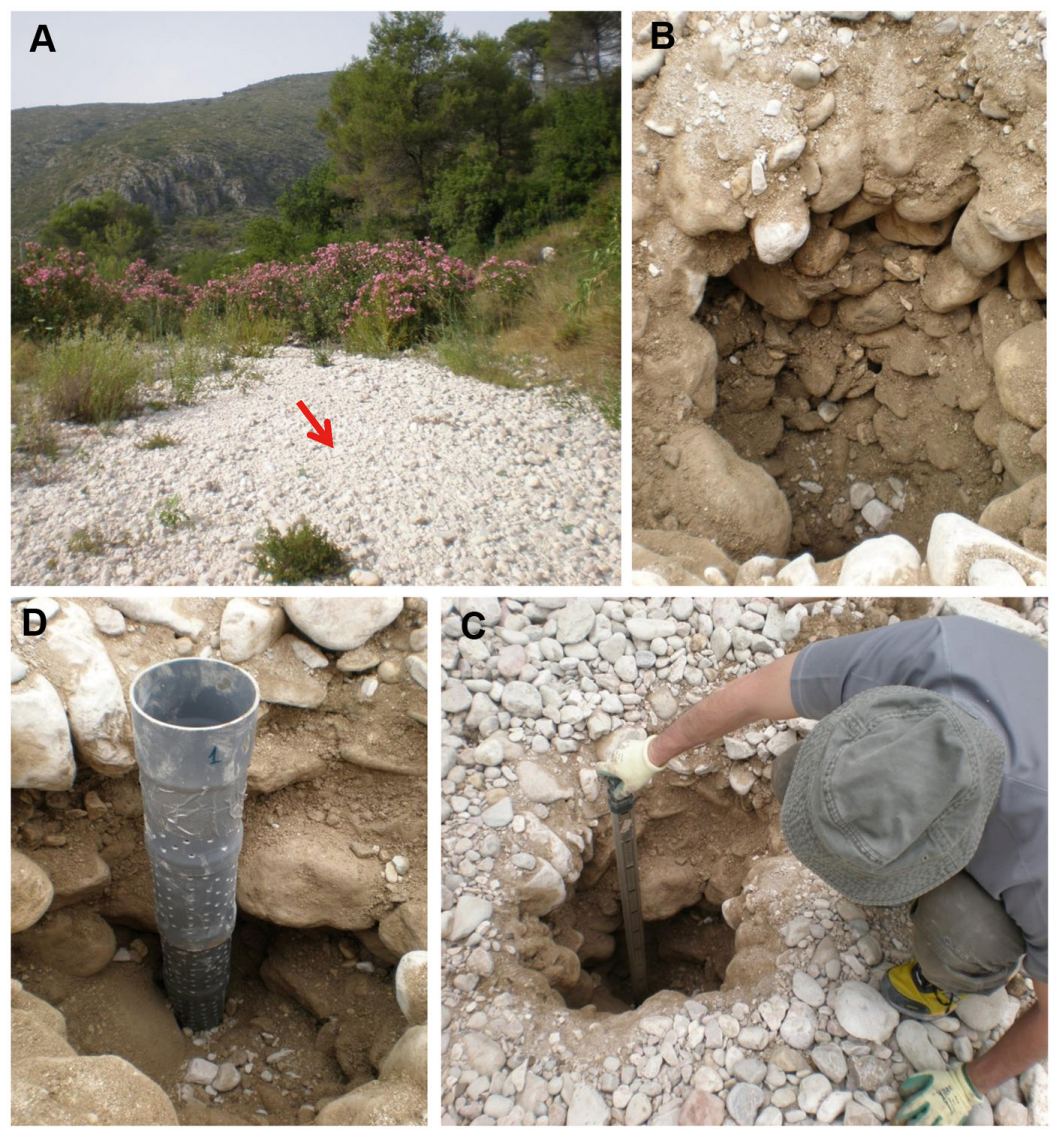
Alluvial MSS. (A) Dry watercourse (Barranc de la Vall de Gallinera). (B, C, D) structure of the substratum.

We propose naming these subterranean spaces located in the alluvial substratum “alluvial MSS”, a hypogean habitat composed of cracks and crevices in the substratum that form among the gravel and variously sized pebbles that constitute the alluvial deposits of dry watercourses (Figure 4). To date, all approaches to the study and recognition of fauna in the substratum of watercourses have focused on the hyporheic zone and, collaterally, with certain adjacent biotopes, some of which might fall under the classification of MSS. The hyporheic zone [60,61] is limited to the substratum of active watercourses, through the interstices of which water circulates and provides a habitat for aquatic species, many of which can be described as stygobionts. In addition, the dynamics of the recent past of these rivers has led to the formation of river terraces beneath which groundwater circulates, constituting aquifers (phreatic biotopes) that may be in contact with the waters of rivers and streams; this forms another type of subterranean water environment [61,] where invertebrate stygobionts are also frequently present. With regard to the MSS that is subject to a fluvial influence and whose origin is unrelated to the dynamics of river currents, Uéno [62] revealed the importance of colluvial deposits and slopes that are more or less directly in contact with the waters of rivers with a moderate current.

The “alluvial MSS” appears in dry watercourses, which appear as scars on the terrain, through which water only flows in times of flood, creating torrential type hydrological regimes. This hydrological dynamic is associated with the Mediterranean climate characteristic of the area, in which maximum rainfall occurs in autumn with a smaller peak in spring, whilst the summer is characterised by a very marked minimum rainfall accompanied by an increase in temperature [24]. Floods occur after irregular, heavy rainfall which starts suddenly and sometimes violently [23,]. However, there are many types of flood, of a diverse nature and intensity [64]. It is of interest to note that those known as *flash floods* [65] whilst not the only type to occur in the areas studied, have a very significant effect since they can even push solid blocks of a diameter of up to twice the height of the water [24,]. These phenomena may be due to different causes, such as summer convective storms, weather fronts and orographic precipitation [67], and often also to the phenomenon known as “cut-off low” [68-70], responsible for numerous catastrophic episodes of flooding.

Since the watercourses in these areas are dry for most of the year, the fissures in the substratum are not permanently waterlogged. However, they retained sufficient water even when water levels were at their lowest for the stones removed during the installation of the SSDs to be damp (unpublished data), and RH_mean_ levels of 80% and RH_min_ levels of 46.5% were recorded for some of the SSDs (Table 1, Barranco de la Cueva de los Corrales). This circumstance facilitates the survival of plants growing in the streambed, provides a refuge for hygrophilous terrestrial fauna and supports the presence of both exclusively and frequently hypogean species, rendering this set of spaces among the stones of alluvial debris a new type of MSS.

### Formation and structure of the “alluvial MSS”

No single factor determines the presence of temporary watercourses in the Eastern Iberian Peninsula. In other regions of the Mediterranean, the presence of temporary watercourses has been described as the result of a synergy of geological, geomorphological and climatic factors [71]. However, in the case of the study area, there is no clear relationship between the presence of such courses and a given geological substrate or a particular rainfall pattern [23]. Furthermore, it should be noted that not all of the Eastern Iberian watercourses are subject to a seasonal regime, as some have a constant flow of water throughout the annual hydrological cycle, either along the entire course or only a part of it, in which groundwater plays an important role in maintaining a base flow [72]. It should also be noted that the presence of dry watercourses is not a feature unique to the Iberian Peninsula. Rather, they appear in numerous regions of the world with very different climates and geology, receiving different names: the “Chapp” in the Gobi, “Laagate” in the Kalahari, “Wadi” in the Maghreb, “Donga” in South Africa, “Nullah” in India, “Fiumare” in Italy, “Arroyo” in the U.S.A. and Mexico, and “Dry Valley” in England [24,].

**Figure 5:**
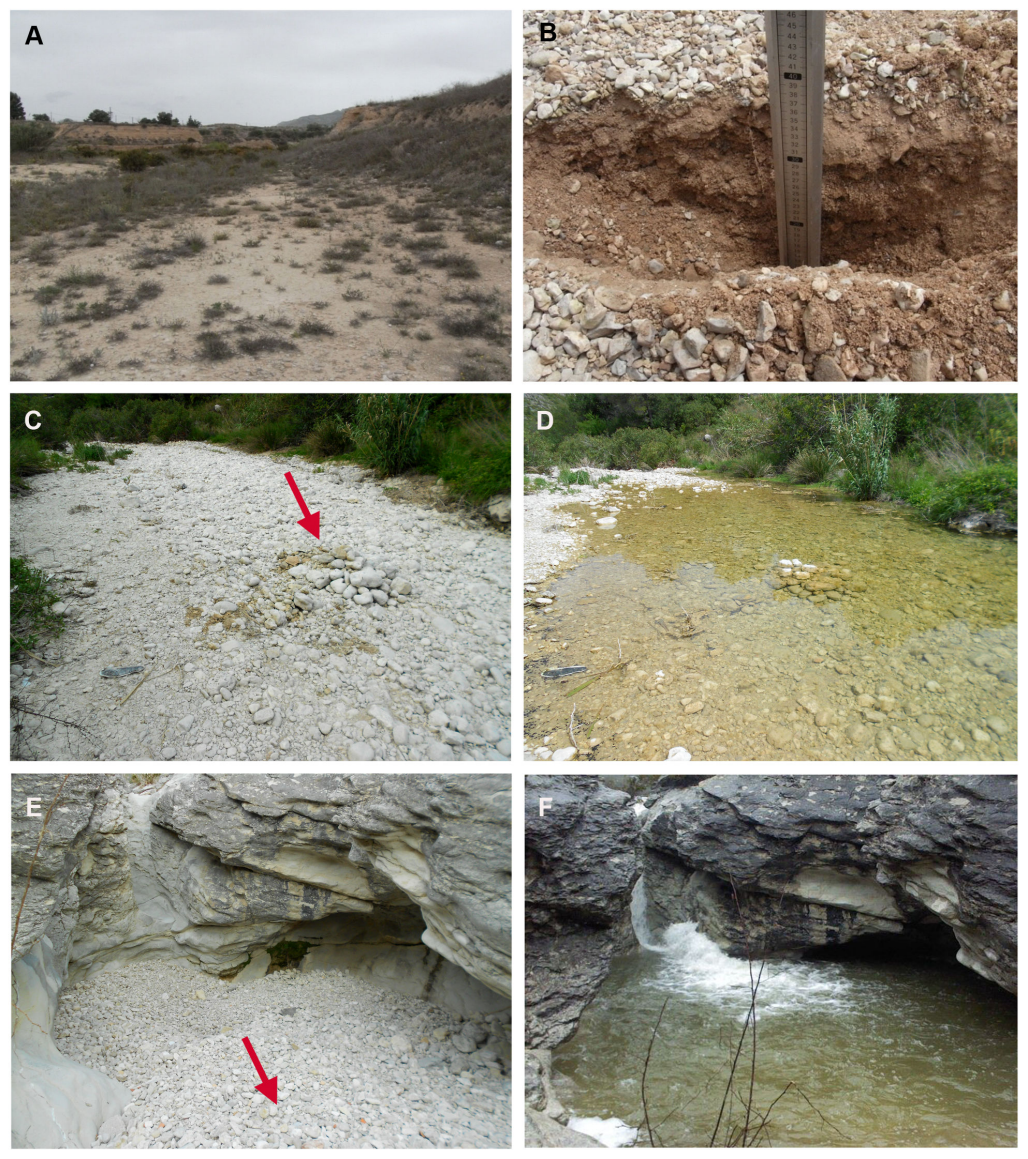
Watercourse with colmated river-bed. (A, B) Rambla de la Torre (Sax, Alicante). Sampled watercouse sections at different moments in time: (C, D) Barranc de la Vall de Gallinera (Subterranean Sampling Device 15); (E, F) Barranc de Famorca (SSD 6).

**Figure 6:**
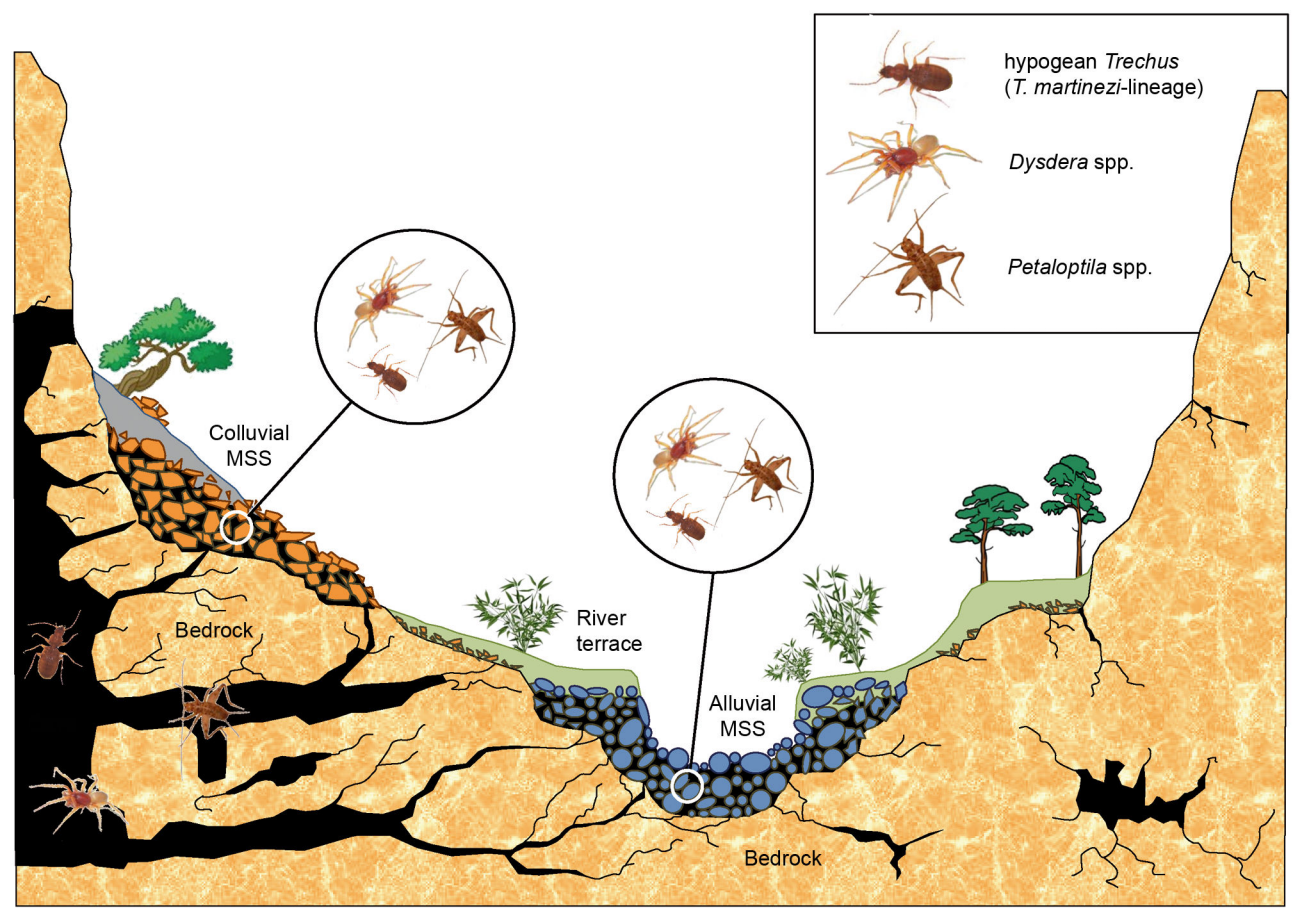
Schematic graph illustrating the connections between hypogean environments, including the “alluvial MSS”. By way of example, three groups of arthropods are shown (*Trechus*: Carabidae, *Dysdera*: Dysderidae, *Petaloptila*: Gryllidae), present in all the subterranean environments in the area (caves, colluvial MSS and “alluvial MSS”).

With regard to the dry watercourses studied, these are known to have a considerable carrying capacity and thus it is very common to see large boulders, pebbles, gravel and sand on the streambed, forming a very heterogeneous group of rocks from different sources [72]. The loss of the water’s inertia results in the rapid and massive sedimentation of the transported materials [74]. This circumstance converts the lower reaches of these dry watercourses (the low, wide channel known as the “*rambla*”) into areas where “alluvial MSS” is unlikely to exist because the interstices between the boulders are generally filled (Figure 5a, b). Our field work revealed the existence of “alluvial MSS” in the upper and middle reaches of these watercourses, where the alluvial debris formed by calcareous stones contained a network of empty spaces which had not been filled by sediments, allowing the movement of fauna between these interstices (Figure 6). However, we cannot assert that dry watercourses in different regions of the world behave in the same way.

Although the “alluvial MSS” is not covered by true soil, it is noteworthy that it may have some degree of insulation from the surface as a result of one phenomenon which occurs with relative frequency in these watercourses. This is the phenomenon known as “armouring” [24,], which occurs when flooding ceases and the carrying capacity of the watercourse decreases. At this point, the coarser materials are deposited, forming protective films or “armour”, and the finest materials filter between and rearrange the interstices. Hence, a thin surface layer of silt, sand and gravel is created between the larger stones close to the surface, which contributes to insulating the MSS to a certain extent (Figure 4b, c, d).

In addition, the vegetation that grows not only on the margins of these dry watercourses but also in their channels, plays an important geomorphological role, proffering mechanical resistance to the water and anchoring the alluvial deposits with their roots. Hence, they provide the “alluvial MSS” with greater structural stability against the drag force of torrential waters and favour sedimentation of the particles carried by the water, thus helping to increase the thickness of these deposits [76]. The dry watercourses studied were mostly colonised by oleander formations, shrubs which are characteristic of dry Mediterranean environments and are adapted to withstand both hot drought and the force of floodwaters. Although the oleander (*Nerium oleander* L.) predominated in these formations, it was often accompanied by various thorny bushes (mostly of the genera *Rubus* L, *Rosa* L. and *Crataegus* L.) and by nitrophilous plants whose growth was favoured by the contribution of organic detritus periodically deposited by floods. When the floods are not very violent or very frequent, it is common for other plants from surrounding plant communities (forest species, mainly) to appear in these streambeds.

The thickness of the alluvial deposits was highly variable, as was the size of the rock fragments of which they were composed, both characteristics being related to the slope and, in general, the orographic and lithological configuration of the site. In some cases, the “alluvial MSS” was no more than 50 cm deep before hitting the bedrock, whilst in other cases its depth exceeded one or one and a half metres (Figure 4).

As already mentioned, the loss of many of the data loggers makes it impossible to generalise the results of the temperature and humidity parameters. Furthermore, the measurements taken corresponded to a depth of between approximately 20 and 30 cm, and it is well known that the physical conditions of temperature and humidity in the substratum change rapidly the further down one goes [77]. However, we can conclude that the “alluvial MSS” is an extremely damp environment, with a RH which is almost constantly in the vicinity of saturation levels. It should be mentioned, however, that the plastic hood in which the data loggers were placed may have produced an overestimation of the RH due to the condensation of water inside. Compared with the external environment, the “alluvial MSS” is very stable as regards temperature [59]. Although the temperature in the MSS showed a pattern (cycle) similar to that of the external environment [59,], values for the mean daily variations in temperature above ground ranged between 10.79-14.78°C, whereas at 20-30 cm deep in the MSS, they ranged between 1.12 and 4.96°C, representing an average of 10°C lower (Figure 3 and Table 2). This is not surprising if we consider that below 50 cm in the substratum, daily temperature variations are usually practically negligible [77], although it may be assumed that in MSS without soil cover, such as the “alluvial MSS”, this depth must be greater [78]. The difference in values between the external environment and the MSS for T_max_ and T_min_ was around 15°C and 5°C on average, respectively. These data show that although there was an appreciable temperature cycle, the MSS had a more modest variation range than the external environment, and suggest that the MSS was a more effective buffer against maximum temperatures than against minimum temperatures [79,].

Therefore, the “alluvial MSS”, is not only similar in structure to other MSSs (a network of subterranean spaces among rocks and stones), but also in its degree of isolation from surface. However, it has some particularities that differentiate it from other MSSs. The origin of the deposit of stones is alluvial, and the isolation from the surface is not due to a soil layer, but to a layer of silt, sand and gravel (Figure 4b, c, d), and it may eventually have disturbances due to floods of variable intensity.

### Ecological importance of the “alluvial MSS”

It is well known that plant and animal species associated with temporary watercourses in arid and semi-arid regions have developed adaptive strategies that allow them to survive water stress [81,]. Watercourses subject to natural spatial and temporal environmental disturbances have been described by Margalef [82] as dynamic evolutionary elements that favour the dynamic processes of species colonisation and expansion [,]. In this paper, we show that this happens not only on the surface and banks of the fluvial network (which includes the edaphic environment), but also in the hypogean environment closely associated with alluvial deposits. In line with the above, the alluvial plains subject to periodic flooding are increasingly being studied as a whole, rather than from an exclusively terrestrial or aquatic perspective [87] As with the “alluvial MSS”, this periodic flooding constitutes a “pulse” that disrupts the dynamic equilibrium these systems had reached before the flood. It is precisely because of these disturbances that it seems difficult to conceive of the existence of terrestrial fauna living in the “alluvial MSS” when, periodically, these channels are completely flooded (Figure 5c-f). However, these apparently catastrophic episodes do not appear to represent an obstacle to the survival of subterranean communities, whose members show very different degrees of adaptation to hypogean life. The proof of this lies in the terrestrial life forms collected in the MSS only days after heavy rainfall. The high speed at which the water flows in the upper and middle sections of these channels may facilitate the retention of large pockets of air within the spaces inside the “alluvial MSS”, and thus, the survival of the terrestrial fauna. The high carrying capacity of these watercourses suggests the possibility that hydrochory events could occur, “seeding specimens” in other sections of the channel suitable for hypogean life [88].

The fauna results obtained show that despite the structural instability of this MSS, the underground spaces in the area studied constituted a habitat that was sufficiently suitable for colonisation both by exclusively hypogean fauna (species only known caves in the area) and frequently hypogean fauna (“troglophile” species sensu Sket [89]). This habitat also acts as a “refuge” for epiedaphic, hygrophilous fauna that are capable of occupying these spaces when necessary to shelter from the seasonal fluctuations in temperature and humidity. Therefore, this subterranean environment must be considered an important habitat due to the role it plays in the conservation of fauna. Furthermore, it represents a new source of information for determining the real biodiversity of sites which, until now, had only been explored above ground and provides data which contribute to improving knowledge of the real distribution of hypogean and epigean species. Lastly, the possibility must also be raised that this new type of MSS may act as an ecological corridor for both hypogean and epiedaphic, hygrophilous fauna.

### Synthesis of characteristics of the “alluvial MSS”

The “alluvial MSS” is a type of terrestrial shallow subterranean habitat that consists of a network of spaces which forms among the alluvial deposits of temporary watercourses, and which may be covered or not by a more or less evolved soil on which some herbaceous or woody plants may manage to grow.

#### Structure

the “alluvial MSS” forms within alluvial deposits of any type of lithology, and has a thickness which ranges from a few decimetres to several metres deep, overlying the bedrock. Among the gravel (a few millimetres in diameter) and rocks (of up to several decimetres in diameter) that comprise this habitat, empty micro and mesovoids can form which facilitate colonisation by, or provide refuge for, terrestrial fauna presenting very diverse ecological preferences.

#### Origin

this habitat is formed as the result of the accumulation of eroded rock fragments (principally pebbles) in dry watercourses, which have been carried there by periodic flooding of the channels.

#### Abiotic factors

it moderates the temperature with respect to the external environment, presents high humidity and, below a certain depth, constitutes an aphotic environment. This habitat is usually affected by sporadic, short-lived and temporary flooding.

#### Energy Sources

nutrients (organic detritus of animal and vegetable origin) transported by floodwater or from the banks by surface runoff, waste products resulting from the metabolism of the animals inhabiting the MSS and plant remains from vegetation growing on the banks and plants growing in the channel itself (grasses and shrubs).

#### Fauna

animals, generally invertebrates and principally arthropods, of different taxa and diverse ecological roles, including epiedaphic, sub-lapidicole, hygrophilous, subterranean (endogean and even hypogean) and ripicolous species. During flooding, it may also contain species which are typically aquatic.

#### Evolution

this subterranean environment appears to be more dynamic and unstable than other types of MSS. Floods encourage the periodic creation and destruction of this habitat’s structure, and it is the uppermost layers of the alluvial deposit which are the most unstable. In addition, the alluvial deposit may also be affected by two different processes that are closely related to the transportation of sediment by water: a periodical washing away of sediments or a process whereby the interstices of the alluvial deposit become filled with sediment.

## Discussion

The streambed of the dry watercourses studied can be included within the category of MSS due to two factors. On the one hand, there are its abiotic characteristics (structure, absence of light, temperature mitigation and high and constant relative humidity compared to the surface, etc.) and on the other hand, there are the biotic characteristics of the fauna found there, although in this case, with some unique elements such as ripicolous fauna. The MSS known to date have consisted of fauna communities similar to those inhabiting caves [90], with hypogean/troglobiont and troglophile fauna living their entire life cycle, or part of it, within these spaces, and with the occasional presence of trogloxene fauna. The beds of the dry watercourses studied contained these three types of fauna, as well as ripicolous elements seeking temporary shelter, justifying the classification of this habitat, together with its structure, as a new type of MSS which we have termed “alluvial MSS”.

A distinguishing characteristic of the “alluvial MSS”, with respect to other MSS, is its dynamic nature, both in terms of its structure and its spatial location. The “alluvial MSS” is unique in that it may suffer periodic or sporadic flooding (Figure 5c-f), depending on the precipitation pattern in the area where it is located. Therefore, in a similar manner to the epikarst, the same spaces can, at different times, serve as a habitat for terrestrial, hypogean, epigean and aquatic fauna. The origins of “alluvial MSS” are similar to the habitats known as Exposed Riverine Sediments (ERS), which have been defined as “exposed, within channel, fluvially deposited sediments (gravels, sands and silts) that lack continuous vegetation cover, whose vertical distribution lies between the levels of bankfull and the typical base flow of the river” [91-93]. Following this definition, there are similarities with the MSS alluvial formation, but there are also two important differences. First of all, in normal conditions, there is no water flowing in the dry riverbeds, which influences the set of biotic and abiotic characteristics of “alluvial MSS” and makes it different from the ERS. Secondly, the alluvial MSS would be similar to the ERS only in its deepest areas and only given the case that there is a network of interstices. Therefore, neither the surface nor the deepest compact sedimentary layers nor the water-filled interstices would be an alluvial MSS in a flowing river. Besides, the “alluvial MSS” has been studied from a subterranean biology perspective, a discipline where the term MSS is widely known, given its usefulness and adequacy for our purpose of definition.

The dry watercourses in the Eastern Iberian Peninsula are subject to an irregular and torrential hydrological regime that affects the fauna and is the driving force behind structural changes in the “alluvial MSS”. As a result, the “alluvial MSS” generally presents as a hypogean environment which is more susceptible to disturbances than other known subterranean environments. Its structural instability, a priori, could hinder the prolonged occupation of fauna which over time has evolved into specialised forms of hypogean life. Consistent with the previous argument, in our study we identified a number of clearly hygrophilous-lucifugous species which until now have only been described in caves and/or colluvial MSS. This circumstance may indicate that the “alluvial MSS” functions as a corridor between peripheral subterranean spaces (colluvial MSS, network of fissures in the bedrock, etc.) which eventually becomes inhabited by this type of fauna from other, more stable subterranean spaces. This suggests that in these areas, which have a Mediterranean climate, the colluvial MSS and the deep subterranean environment do not necessarily constitute isolated pockets of subterranean biodiversity. The “alluvial MSS” could facilitate the interconnection of all these subterranean spaces, since it is distributed throughout the length of the river basins dividing the different karst massifs. To some extent, this calls into question the classic notion that rivers constitute geographical barriers for hypogean terrestrial fauna, as cited by other authors [30]. In this sense, the “alluvial MSS” may be considered to form a connection between nearby karst reliefs (Figure 6). An important detail to bear in mind is that watercourses become effective barriers for this type of fauna when their hydrological regime is constant, or when they flow over a lithology which is unsuitable for hypogean life, such as clay/marl deposits.

However, a much wider view must be taken of the “alluvial MSS” than that provided by the classic perspective on subterranean biology. The stenohygrobic epigean fauna of the Quaternary associated with Eastern Iberian rivers has seen its survival compromised to a large extent by the climate changes which have occurred in recent millennia [95,]. One of the factors which has contributed to the survival of this kind of fauna has been the possibility of taking refuge in the interstices of the soil/substratum, where humidity and temperature are more suitable, as indicated by Růžička [96] when stating that “fauna migrations, caused by great climatic changes are important factors promoting the colonization of the subterranean environment”. The remarkable diversity of epiedaphic stenohygrobic fauna inhabiting the “alluvial MSS” renders these spaces subterranean oases of life, so that in areas that are more or less xeric, species which have largely or completely disappeared from epigean habitats continue to exist. Such is the case of the ripicolous species that survive in the “alluvial MSS” but are absent for most of the year from the streambed of these “dry rivers”. Since the “alluvial MSS” is distributed along the length of dendritic shaped routes formed by the streambeds of watercourses, and presents high humidity even in summer periods, this hypogean habitat could act as a wildlife corridor. This role could be intensified by sporadic episodes of hydrochory, as occurs with some endogean beetles in the upper reaches of streams [88], where torrential river floods carrying large amounts of sediment also transport some of the fauna that inhabits it.

The abundance of *dry watercourses* globally, leads us to conclude with the following observations:

1 It is possible that in addition to being a habitat for a very diverse range of fauna, the “alluvial MSS” may function as a corridor for hypogean fauna between a priori isolated karst massifs around the Mediterranean and elsewhere.
2 In addition, we wish to emphasise the ecological importance of this type of environment as regards providing a suitable habitat for the hygrophilous fauna now largely absent from the surface due to climate change and anthropogenic factors that have increased environmental xericity. This leads us to raise a third question.
3 Mediterranean river sites shelter large amounts of “hidden biodiversity”, rendering them areas of special interest for hygrophilous species associated with the soil. Further research is required in order to understand their role in the conservation of fauna communities affected by climate change, both in the present and in the past.

## Supporting Information

Table S1 **Arthropoda species collected in the alluvial MSS.** So far, a total of 133 species have been identified, distributed among the 16 sampled localities in the province of Alicante (Eastern Spain): 1, Barranco de la Cueva de los Corrales; 2, Barranc dels Ports; 3, Barranc del Xarquet; 4, Barranc de Sacanyar; 5, Río Bolulla; 6, Barranc de Famorca; 7, Barranc de Almadich; 8, Barranc de Malafí; 9, Río Castells; 10, Barranc de Masserof; 11, Río Xaló; 12, Barranc d’Alcalà; 13, Barranc de Turrubanes; 14, Barranc de Cocons; 15, Barranc de la Vall de Gallinera; 16, Barranc de la Vall de Gallinera.
(DOC)
