# Supplementary material for: The “Alluvial Mesovoid Shallow Substratum”, a New Subterranean Habitat
Source: PLoS One. 2013 Oct 4;8(10):e76311. doi: 10.1371/journal.pone.0076311 (PMC3790681; doi:10.1371/journal.pone.0076311)
Supplement: Table S1 — Arthropoda species collected in the alluvial MSS. So far, a total of 133 species have been identified, distributed among the 16 sampled localities in the province of Alicante (Eastern Spain): 1, Barranco de la Cueva de los Corrales; 2, Barranc dels Ports; 3, Barranc del Xarquet; 4, Barranc de Sacanyar; 5, Río Bolulla; 6, Barranc de Famorca; 7, Barranc de Almadich; 8, Barranc de Malafí; 9, Río Castells; 10, Barranc de Masserof; 11, Río Xaló; 12, Barranc d’Alcalà; 13, Barranc de Turrubanes; 14, Barranc de Cocons; 15, Barranc de la Vall de Gallinera; 16, Barranc de la Vall de Gallinera. (DOC) [file pone.0076311.s001.doc]

**Appendix A Online.** Arthropoda species collected in the alluvial MSS. So far, a total of 133 species have been identified, distributed among the 16 sampled localities in the province of Alicante (Eastern Spain): 1, Barranco de la Cueva de los Corrales; 2, Barranc dels Ports; 3, Barranc del Xarquet; 4, Barranc de Sacanyar; 5, Río Bolulla; 6, Barranc de Famorca; 7, Barranc de Almadich; 8, Barranc de Malafí; 9, Río Castells; 10, Barranc de Masserof; 11, Río Xaló; 12, Barranc d’Alcalà; 13, Barranc de Turrubanes; 14, Barranc de Cocons; 15, Barranc de la Vall de Gallinera; 16, Barranc de la Vall de Gallinera.

| **Taxa** | | **Presence of taxa in each locality (1-16) of “alluvial MSS”** | | | | | | | | | | | | | | | |
| --- | --- | --- | --- | --- | --- | --- | --- | --- | --- | --- | --- | --- | --- | --- | --- | --- | --- |
|  |  | **1** | **2** | **3** | **4** | **5** | **6** | **7** | **8** | **9** | **10** | **11** | **12** | **13** | **14** | **15** | **16** |
| ARACHNIDA | Araneae |  |  |  |  |  |  |  |  |  |  |  |  |  |  |  |  |
|  | *Agroeca inopina* O.P. Cambridge, 1886 | − | − | − | − | − | − | − | − | − | − | − | − | X | − | − | − |
|  | *Dysdera* cf. *erythrina* (Walckenaer, 1802) | − | − | − | − | − | − | − | − | − | − | − | X | − | − | − | X |
|  | *Dysdera espanoli* Ribera & Ferrández, 1986 | X | X | − | − | − | − | − | − | − | − | − | − | − | − | − | X |
|  | *Dysdera scabricula* Simon, 1882 | − | − | − | X | − | − | − | X | X | − | − | − | X | − | − | − |
|  | *Dysdera* n. sp. | − | − | − | − | − | − | − | − | − | − | − | − | − | X | X | − |
|  | *Ero* sp. | − | − | − | − | − | − | − | − | − | X | − | − | − | − | − | − |
|  | *Gnaphosa alacris* Simon, 1878 | − | − | − | − | − | − | − | − | − | − | X | − | − | − | − | − |
|  | *Harpactea* n. sp. | − | X | − | − | X | − | − | − | − | − | − | − | − | − | − | − |
|  | *Kaemis* n. sp. | − | − | − | − | − | − | X | − | − | − | − | − | − | − | − | − |
|  | *Lepthyphantes* (*sensu lato*) spp. | X | X | − | X | X | X | X | X | − | X | − | − | X | − | − | X |
|  | *Lessertia dentichelis* (Simon, 1884) | − | − | − | − | X | − | − | − | X | − | X | X | − | − | − | − |
|  | *Loxosceles* sp. | − | X | − | − | − | − | − | − | − | − | − | − | − | − | − | − |
|  | *Malthonica picta* (Simon, 1870) | − | − | − | − | − | − | X | − | − | − | − | − | − | − | − | − |
|  | *Micaria* sp. | − | − | − | − | − | − | − | − | − | X | − | − | X | − | − | − |
|  | *Pardosa* cf. *tatarica* (Thorell, 1875) | − | − | X | − | − | − | X | − | X | − | X | − | − | − | − | − |
|  | *Scytodes thoracica* (Latreille, 1802) | − | − | − | X | − | − | − | − | − | − | − | − | − | − | − | − |
|  | *Silhouettella loricatula* (Roewer, 1942) | − | − | − | X | − | − | − | − | X | − | − | − | − | − | − | − |
|  | *Steatoda triangulosa* (Walckenaer, 1802) | − | − | − | − | − | − | − | − | − | − | X | − | − | − | − | − |
|  | *Tegenaria fuesslini* Pavesi, 1873 | X | X | − | − | − | − | − | − | − | − | − | − | − | − | − | − |
|  | *Tegenaria levantina* Barrientos, 1981 | X | X | − | − | − | − | − | − | X | − | − | − | − | − | − | − |
|  | *Trachyzelotes fuscipes* (L. Koch, 1866) | X | − | − | − | − | − | X | − | X | X | X | − | − | − | X | X |
|  | *Zelotes* sp. | − | X | − | − | X | − | − | − | X | − | X | − | X | − | − | X |
|  | *Zodarion costablancae* Bosmans, 1994 | − | − | − | − | − | − | − | − | X | − | X | − | − | − | − | − |
|  | Opiliones |  |  |  |  |  |  |  |  |  |  |  |  |  |  |  |  |
|  | *Dicranolasma soerensenii* Thorell, 1876 | − | − | − | X | X | − | X | X | − | X | − | − | − | − | − | − |
|  | *Trogulus lusitanicus* Giltai, 1931 | − | − | − | X | − | − | X | X | − | X | − | − | X | X | − | − |
|  | Acari |  |  |  |  |  |  |  |  |  |  |  |  |  |  |  |  |
|  | *Allogalumna parva* (Berlese, 1916) | − | − | − | − | X | − | − | − | − | − | − | − | − | − | − | − |
|  | *Arthrodamaeus mediterraneus* Subías, Arillo & Subías, 1997 | − | − | − | − | − | − | − | − | − | − | − | − | − | − | − | X |
|  | *Carabodes (Klapperiches)* sp. | − | − | X | − | − | − | − | − | − | − | − | − | − | − | − | − |
|  | *Cepheus pegazzanoae* Bernini & Nannelli, 1982 | − | − | − | − | − | − | − | − | − | − | − | − | X | − | − | − |
|  | *Ceratoppia bipilis* (Hermann, 1804) | − | − | − | − | X | − | − | − | − | − | − | − | − | − | − | − |
|  | *Chamobates (Xiphobates) sergienkoae* Shaldybina, 1980 | − | − | X | − | − | − | − | − | − | − | − | − | − | − | − | − |
|  | *Damaeus (Epidamaeus) berlesei* Michael, 1908 | − | X | − | − | − | − | − | − | − | − | − | − | − | − | − | − |
|  | *Dometorina plantivaga* (Berlese, 1895) | − | − | − | − | − | − | − | X | − | − | − | X | − | − | − | − |
|  | *Epilohmannia cylindrica* (Berlese, 1904) | − | − | − | − | − | − | − | − | − | − | X | − | − | − | − | − |
|  | *Epilohmannia styriaca* Schuster, 1960 | − | − | − | − | − | − | − | − | − | − | − | − | − | − | − | X |
|  | *Eupelops acromios* (Hermann, 1804) | − | − | − | − | − | − | − | X | − | − | − | X | − | − | − | − |
|  | *Galumna* cf. *flagellata* Willmann, 1925 | − | − | − | − | X | − | − | − | − | − | − | − | − | − | − | − |
|  | *Galumna* cf. *setigera* Mihelčič, 1956 | − | − | − | − | − | − | X | − | − | X | X | − | − | − | − | − |
|  | *Galumna tarsipennata* Oudemans, 1914 | − | − | − | − | − | − | − | − | − | − | − | X | − | − | − | − |
|  | *Globozetes longipilus* Sellnick, 1928 | − | X | − | − | − | − | − | − | − | − | − | − | − | − | − | − |
|  | *Gustavia oceanica* Pérez-Íñigo, 1987 | − | − | − | − | X | − | − | X | − | − | − | − | − | − | − | − |
|  | *Hemileius eperezinigoae* Subías, 2010 | − | − | − | − | X | − | − | − | − | − | − | X | − | − | − | − |
|  | *Hermanniella dolosa* Grandjean, 1931 | − | − | − | − | X | − | − | − | − | − | − | − | X | − | − | − |
|  | *Humerobates rostrolamellatus guadarramicus* Pérez-Íñigo, 1972 | − | − | − | − | X | − | − | − | − | − | − | X | − | − | − | − |
|  | *Lucoppia burrowsi* (Michael, 1890) | − | − | − | − | X | − | − | − | − | − | X | X | − | − | X | − |
|  | *Neoliodes globosus* (Subías & Gil-Martín, 1990) | − | − | − | − | − | − | − | − | − | − | X | − | − | − | − | − |
|  | *Nothrus anauniensis* Canestrini & Fanzago, 1876 | − | − | − | − | − | − | X | − | − | − | − | − | − | − | − | − |
|  | *Oppia denticulata* (G. & R. Canestrini, 1882) | − | − | − | − | X | − | − | − | − | − | − | − | − | − | − | − |
|  | *Oribatula (Oribatula) tibialis allifera* Subías, 2000 | − | − | − | − | − | − | X | − | − | − | X | X | − | − | − | − |
|  | *Oribatula (Oribatula) tibialis tibialis* (Nicolet, 1855) | X | − | X | − | X | − | X | X | − | − | − | − | X | − | − | − |
|  | *Oribatula (Zygoribatula) frisiae* (Oudemans, 1900) | − | − | X | − | − | − | X | − | − | − | − | − | − | − | − | − |
|  | *Phauloppia lucorum* (Koch, 1841) | − | − | − | − | − | − | − | − | − | − | X | − | − | − | − | − |
|  | *Phthiracarus laevigatus* (Koch, 1841) | − | − | − | − | − | − | − | X | − | − | − | − | X | − | − | − |
|  | *Phthiracarus longulus* (Koch, 1841) | − | − | − | − | X | − | − | − | − | − | − | − | − | − | − | − |
|  | *Ramusella (Insculptoppiella) elongata* (Paoli, 1908) | − | − | X | − | X | − | − | − | − | − | − | − | − | − | − | − |
|  | *Scheloribates barbatulus* Mihelčič, 1956 | − | − | − | − | − | − | − | − | − | − | X | − | − | − | − | − |
|  | *Scheloribates laevigatus angustirostris* Mihelčič, 1957 | − | − | X | − | − | − | − | − | − | − | − | − | X | − | − | − |
|  | *Scutovertex pictus* Kunst, 1959 | − | − | X | − | − | − | − | − | − | − | − | − | − | − | − | − |
|  | *Tectocepheus minor* Berlese, 1903 | − | − | X | − | − | − | − | − | − | − | − | − | − | − | − | − |
|  | *Topobates holsaticus* Weigmann, 1969 | − | − | X | − | − | − | − | − | − | − | − | X | − | − | − | − |
|  | *Xenillus clavatopilus* Mihelčič, 1966 | − | − | − | − | X | − | − | − | − | − | − | − | X | − | − | − |
|  | *Xenillus tegeocranus* (Hermann, 1804) | − | − | − | − | − | − | − | − | − | − | − | − | − | − | − | X |
|  | *Zetorchestes grandjeani* Krisper, 1987 | − | − | − | − | − | − | − | − | − | − | − | − | X | − | − | − |
| CRUSTACEA | Isopoda |  |  |  |  |  |  |  |  |  |  |  |  |  |  |  |  |
|  | *Anaphiloscia simoni* Racovitza, 1907 | − | − | X | − | − | − | − | − | − | − | − | − | − | − | − | − |
|  | *Armadillo officinalis* Duméril, 1816 | − | − | X | − | − | − | − | − | − | − | − | − | X | − | − | − |
|  | *Chaetophiloscia elongata* (Dollfus, 1884) | − | − | − | − | − | − | − | − | − | X | − | − | − | − | − | − |
|  | *Porcellio incanus* Budde-Lund, 1879 | − | − | − | − | X | − | − | − | − | X | − | − | X | − | − | − |
|  | *Porcellionides pruinosus* (Brandt, 1833) | − | − | − | − | − | − | − | − | − | X | − | − | − | − | − | − |
|  | *Trichoniscus provisorius* Racovitza, 1908 | − | − | X | − | X | − | − | − | − | X | − | − | − | − | − | − |
|  | *Trichoniscus* sp. | − | − | − | − | − | − | − | − | − | − | − | − | X | − | − | − |
| MYRIAPODA | [Lithobiomorpha](http://es.wikipedia.org/wiki/Lithobiomorpha) |  |  |  |  |  |  |  |  |  |  |  |  |  |  |  |  |
|  | *Lithobius castaneus* Newport, 1844 | X | X | X | − | X | − | − | X | − | X | − | − | − | X | − | X |
|  | *Lithobius lusitanicus* Verhoeff, 1925 | − | − | − | − | − | − | − | − | X | − | X | − | − | − | − | X |
|  | *Lithobius* sp. | − | − | − | − | − | X | − | − | − | − | − | − | X | − | − | − |
|  | [Scolopendromorpha](http://www.faunaeur.org/full_results.php?id=11606) |  |  |  |  |  |  |  |  |  |  |  |  |  |  |  |  |
|  | *Cryptops hispanus* Brolemann, 1920 | − | − | − | − | X | − | − | − | − | − | − | − | − | − | − | − |
|  | *Scolopendra cingulata* Latreille, 1789 | − | − | − | − | − | − | − | X | X | − | X | − | − | − | − | − |
|  | *Scolopendra oraniensis* H. Lucas, 1846 | X | − | − | − | − | − | − | − | − | − | − | − | − | − | − | − |
|  | *Theatops erythrocephala* (C.L.Koch, 1847) | − | − | − | − | − | X | X | X | − | − | − | − | − | − | − | X |
|  | Scutigeromorpha |  |  |  |  |  |  |  |  |  |  |  |  |  |  |  |  |
|  | *Scutigera coleoptrata* (Linnaeus, 1758) | − | − | X | − | − | X | − | X | X | X | X | − | X | − | − | X |
|  | Julida |  |  |  |  |  |  |  |  |  |  |  |  |  |  |  |  |
|  | *Ommatoiulus* sp. | − | − | − | − | − | − | − | − | − | − | X | − | − | − | − | − |
|  | [Polydesmida](http://es.wikipedia.org/wiki/Lithobiomorpha) |  |  |  |  |  |  |  |  |  |  |  |  |  |  |  |  |
|  | *Brachydesmus proximus* Latzel, 1889 | − | − | − | − | − | − | − | − | − | − | X | − | − | X | − | − |
|  | *Brachydesmus superus* Latzel, 1884 | − | − | − | − | − | − | − | − | − | X | − | − | − | − | − | X |
|  | *Stosatea capolongoi* Strasser, 1971 | − | − | − | − | X | X | X | X | − | X | − | − | X | − | − | − |
|  | Callipodida |  |  |  |  |  |  |  |  |  |  |  |  |  |  |  |  |
|  | *Cyphocallipus excavatus* Verhoeff, 1909 | X | X | − | − | − | − | − | X | − | X | − | − | − | − | − | − |
|  | Glomerida |  |  |  |  |  |  |  |  |  |  |  |  |  |  |  |  |
|  | *Glomeris* sp. | − | − | − | − | − | − | − | − | − | − | − | − | − | − | X | − |
| HEXAPODA | [Collembola](http://es.wikipedia.org/wiki/Lithobiomorpha) |  |  |  |  |  |  |  |  |  |  |  |  |  |  |  |  |
|  | *Ballistura palustris* (Cassagnau, 1959) | − | X | − | − | − | − | − | − | − | − | − | − | − | − | − | − |
|  | *Ballistura schoetti* (Dalla Torre, 1895) | − | − | − | − | − | − | − | − | − | − | X | − | − | − | − | − |
|  | *Bilobella aurantiaca* (Caroli, 1910) | − | − | − | − | − | − | − | − | − | − | X | − | − | − | − | − |
|  | *Caprainea marginata* (Schoett, 1893) | − | − | − | − | − | − | − | − | − | − | − | − | − | − | X | − |
|  | *Ceratophysella engadinensis* (Gisin, 1949) | − | − | X | − | − | − | − | − | − | − | X | − | − | − | − | − |
|  | *Ceratophysella gibbosa* (Bagnall, 1940) | − | − | − | − | − | − | − | − | − | − | X | − | − | − | − | − |
|  | *Dicyrtoma fusca* (Lubbock, 1873) | − | − | − | − | X | − | − | − | − | − | X | − | − | − | − | − |
|  | *Friesea* sp. | − | − | X | − | − | − | − | − | − | − | − | − | − | − | − | − |
|  | *Gisinurus malatestai* Dallai, 1970 | − | X | − | − | − | − | − | − | − | − | − | − | − | − | − | − |
|  | *Heteromurus major* (Moniez, 1889) | − | X | − | − | − | − | − | − | − | − | X | − | − | − | − | − |
|  | *Heteromurus nitidus* (Templeton, 1835) | X | − | − | − | X |  | − | − | − | − | − | − | X | − | − | − |
|  | *Hypogastrura affinis* (Lucas, 1846) | − | − | − | − | − | − | X | − | − | − | − | − | − | − | − | − |
|  | *Hypogastrura manubrialis* (Tullberg, 1869) | − | − | − | − | − | − | − | − | − | − | X | − | − | − | − | − |
|  | *Isotomurus* sp. | − | − | − | − | X | − | − | − | − | − | − | − | − | − | X | − |
|  | *Lepidocyrtus flexicollis* Gisin, 1965 | X | X | − | − | − | − | − | − | − | X | X | − | X | X | − | − |
|  | *Lepidocyrtus* sp. | X | X | − | − | X | − | X | − | − | − | − | − | − | − | − | − |
|  | *Neelus murinus* Folsom, 1896 | − | − | − | − | − | − | X | − | − | X | − | − | − | − | − | − |
|  | *Neosminthurus natalicia* Ellis, 1974 | − | − | − | − | X | − | − | − | − | − | − | − | − | − | − | − |
|  | *Orchesella bifasciata* Nicolet, 1842 | − | − | − | − | − | − | − | − | − | − | − | − | X | − | − | − |
|  | *Parisotoma notabilis* (Schaeffer, 1896) | − | − | X | − | − | − | − | − | − | − | − | − | − | − | − | − |
|  | *Protaphorura prolata* (Gisin, 1956) | − | − | − | − | − | − | − | − | − | − | X | − | − | − | − | − |
|  | *Pseudachorudina meridionalis* (Bonet, 1929) | − | − | X | − | − | − | − | − | − | − | − | − | − | − | − | − |
|  | *Pseudachorutes* sp. | − | − | − | − | − | − | − | − | − | − | − | − | X | − | − | − |
|  | *Pseudisotoma* sp. | − | − | X | − | − | − | − | − | − | − | − | − | − | − | − | − |
|  | *Pseudosinella* sp. | − | X | − | − | − | − | − | − | − | − | − | − | − | − | − | − |
|  | *Seira* sp. | − | − | − | − | X | − | − | − | − | − | − | − | X | − | − | X |
|  | *Sminthurus viridis* (Linnaeus, 1758) | − | − | − | − | X | − | − | − | − | − | − | − | − | − | − | − |
|  | *Superodontella vallvidrerensis* (Selga, 1966) | − | − | X | − | − | − | − | − | − |  |  |  |  |  |  |  |
|  | *Xenylla maritima* Tullberg, 1869 | − | − | X | − | − | − | − | − |  |  |  |  |  |  |  |  |
|  | Diplura |  |  |  |  |  |  |  |  |  |  |  |  |  |  |  |  |
|  | *Campodea fragilis* Meinert, 1865 | − | − | − | − | − | − | − | X | − | − | − | − | − | − | − | − |
|  | *Campodea grassii* Silvestri, 1912 | − | − | X | − | − | − | − | X | − | − | − | − | − | − | − | − |
|  | Orthoptera |  |  |  |  |  |  |  |  |  |  |  |  |  |  |  |  |
|  | *Petaloptila (Petaloptila) aliena* (Brunner-Wattenwyl, 1882) | − | X | − | X | X | − | X | − | X | − | X | X | X | X | − | − |
|  | *Petaloptila (Zapetaloptila)bolivari* (Cazurro, 1888) | − | − | − | X | X | − | X | − | X | − | X | − | − | − | − | − |
|  | Coleoptera |  |  |  |  |  |  |  |  |  |  |  |  |  |  |  |  |
|  | *Abacetus (Astigis) salzmanni* (Germar, 1824) | − | − | − | − | − | − | − | − | − | − | X | − | − | − | X | X |
|  | *Bembidion (Ocyturanes) martachemai* (Toribio, 2002) | X | X | X | X | X | X | X | X | X | X | X | X | X | X | − | − |
|  | *Lamprohiza* sp. | − | X | − | X | X | X | X | X | − | X | − | − | − | − | − | − |
|  | *Nyctophila reichii* (Jacquelin du Val, 1859) | − | X | − | − | − | − | − | − | X | X | X | − | X | − | − | − |
|  | *Ocys harpaloides* (Audinet-Serville, 1821) | − | − | − | − | − | − | − | − | X | − | X | − | X | − | − | − |
|  | *Platyderus* sp. | X | − | − | − | X | − | X | − | − | X | − | − | − | − | − | − |
|  | *Porotachys bisulcatus* (Nicolaï, 1822) | − | − | − | X | X | − | − | − | − | − | − | − | X | X | − | − |
|  | *Speonemadus escalerai* (Uhagon, 1898) | X | X | − | − | − | − | − | − | − | − | − | − | − | − | − | − |
|  | *Tachyura (Tachyura) parvula* (Dejean, 1831) | − | − | X | − | − | − | − | − | − | − | − | − | − | − | − | − |
|  | *Trechus obtusus* Erichson, 1837 | − | − | − | X | − | − | − | X | − | X | − | − | − | − | − | − |
|  | *Trechus* n. sp. | − | − | − | X | X | − | − | X | − | − | − | − | X | − | − | − |
|  | Hymenoptera |  |  |  |  |  |  |  |  |  |  |  |  |  |  |  |  |
|  | *Camponotus (Tanaemyrmex) sylvaticus* (Olivier, 1792) | − | − | − | − | X | − | − | − | − | − | − | − | − | − | − | − |
|  | *Crematogaster scutellaris* (Olivier, 1792) | − | − | − | − | − | X | − | − | − | − | − | − | − | − | − | − |
|  | *Diplorhoptrum latro* (Forel, 1894) | − | − | − | − | − | − | − | − | − | − | − | − | X | − | − | − |
|  | *Diplorhoptrum robusta* (Bernard, 1952) | − | − | − | − | X | − | − | − | − | − | − | − | − | − | − | − |
|  | *Pheidole pallidula* (Nylander, 1849) | − | X | − | − | X | − | − | − | − | − | − | − | − | − | X | X |
